# Associations between intestinal parasitic infections, anaemia, and diarrhoea among school aged children, and the impact of hand-washing and nail clipping

**DOI:** 10.1186/s13104-019-4871-2

**Published:** 2020-01-02

**Authors:** Mahmud Abdulkader Mahmud, Mark Spigt, Afework Mulugeta Bezabih, Geert-Jan Dinant, Roman Blanco Velasco

**Affiliations:** 1 Medical Microbiology and Immunology Department, Biomedical Division, School of Medicine, Mekelle University, PO. Box: 1871, Mekelle, Ethiopia; 2 Department of Family Medicine, CAPHRI School for Public Health and Primary Care, Maastricht University, Maastricht, The Netherlands; 3 School of Public Health, College of Health Sciences, Mekelle University, Mekelle, Ethiopia; 4 Department of Surgery, School of Medicine, Alcala University, Madrid, Spain

**Keywords:** Intestinal parasitic infections, Anaemia, Diarrhoea, Hand-washing, School-aged children

## Abstract

**Objective:**

In marginalized setting, under-nutrition and illnesses due to infectious agents create a vicious circle. In our previous study, we reported that easy-to-do hand hygiene interventions were effective in preventing intestinal parasite infections (IPIs) and reduce the rate of anaemia among school-aged children. The aim of this study was to assess the pattern of associations between IPIs, anaemia and diarrhoea among the school-aged children and to explore if the observed impact of hand-washing and nail clipping interventions in our findings was similar across children with different baseline demographic and disease characteristics. The study was based on the analysis of data that was collected during the randomized controlled trial and hence have used the same study participants and study area.

**Results:**

Children with IPIs had a much higher chance of also being anaemic (AOR 2.09, 95% CI 1.15–3.80), having diarrhoea (AOR 2.83, 95% CI 1.57–5.09), and vice versa. Anaemia and diarrhoea were very strongly related (AOR 9.62, 95% CI 5.18–17.85). Overall, hand-washing with soap at key times and weekly nail clipping were efficacious in preventing intestinal parasite re-infection among children despite the differences in baseline demographic characteristics.

*Trial registration*: NCT01619254 (June 09/2012)

## Introduction

In resource‐poor settings, under‐nutrition and illnesses due to infectious diseases are highly prevalent and closely interlinked. Nutritional deficiencies predispose people to infection, and infections lead to nutritional deficiencies which further reduce resistance to new infections [1–3]. Children are especially susceptible to the deleterious effects of under‐nutrition [4] and infections [5] in developing settings.

In Ethiopia, similar to many developing countries, IPIs [6, 7] and anaemia [6, 8] are common among school‐aged children. This suggests that school‐aged children in Ethiopia may be vulnerable to the cyclical ill‐effects of anaemia and illnesses due to parasitic infections. However, there is insufficient evidence regarding the associations of IPIs, anaemia and diarrhoea among these population groups in the country.

Remarkable heterogeneity is documented in the distribution patterns of IPIs [9]. Several demographic [10], socioeconomic [11], and environmental [12] factors influence the distribution patterns of IPIs within a community. These factors can create different distributions of susceptibility to re‐infection by IPIs among a population, and hence, different groups of children may be at different risks of parasitic re‐infection.

A randomised controlled trial revealed a significant impact of regular hand‐washing with soap on the prevention of parasitic re‐infection and anaemia among children [13]. It is unknown if certain characteristics of the children also determined the effects of hand‐washing. One could imagine, for example, that in areas where parasites are distributed by polluted water, that hand‐washing would not be effective in preventing parasitic infections. We set out to explore if the effect of hand‐washing and nail clipping interventions in our findings [13] was similar across the study population despite their different characteristics.

## Main text

### Methods

#### Description of the trial

In our previous randomized controlled trial [13], we reported that easy-to-do hand washing interventions were effective in preventing intestinal parasite infections (IPIs) and reduce the rate of anaemia among school-aged children. This study was carried out on the analysis of data collected during the randomized controlled trial with the objective of assessing whether the observed efficacy of the easy-to-do hand washing interventions was universally effective to prevent infection among the same school‐aged children with different socio-demographic baseline characteristics. Moreover, we aimed to explore whether infection and malnutrition were interlinked and hence the infection prevention interventions used in the previous study would break the vicious cycle of infection and malnutrition in such marginalized areas for the long-term health benefits of this population group.

#### Data collection procedure

##### Baseline data

briefly, demographics (child age, gender, latrine use, maternal age, family size, family drinking water source, and living house ownership), socio economic (maternal education) and pre‐existing disease characteristics (IPIs, anaemia, and thinness) were included for this analysis from data collected during the previous randomized controlled trial [13].

##### Parasitological analysis

Following 6 months follow‐up, fresh stool specimens were collected from the study subjects. Stool specimens were analysed using direct saline wet mount, formalin ethyl‐acetate concentration technique [14] and the Kato‐Katz technique [15]. A child was classified as re‐infected if an infection was detected by any methods used. Sub‐samples of stool smears, comprising 10% of the total, were re‐examined for quality control purposes.

##### Diarrhoea incidence

Data on self‐reported diarrhoeal episodes were collected using a separate questionnaire on a weekly basis during the study period. Diarrhoea was defined as the passage of three or more loose or liquid stools per day [16].

##### Anthropometry

Anthropometric measurements were taken at the start and the end of the follow‐up in duplicate and the average of the two measurements was recorded. Portable weight scales and locally made stadiometers with a sliding headpiece were used to measure weight (to the nearest 0.1 kg) and height (to the nearest 0.1 cm), respectively. Each child was weighed with minimal clothing and barefooted. The weighing scales were calibrated using standard calibration weights of 5 kg iron bars. Height measurements were taken with children faced forwards, barefooted with feet flat and together, and their heels and back against the rod. Anthropometric measurements were converted into BMI‐for‐age Z scores using WHO AnthroPlus software, version 1.0.4 (WHO Anthro 2007, WHO, Geneva, Switzerland). Children below − 2Z scores for BMI‐for‐age were classified as thin.

#### Statistical analysis

Statistical analysis was computed using SPSS for Windows version 16.0 (Chicago, USA). Associations between post‐intervention IPIs, anaemia, and diarrhoea were analyzed using binary and multivariate logistic regression models by odds ratios (OR) and 95% confidence intervals (CI). The impact of hand‐washing with soap and nail clipping on intestinal parasite re‐infection across children with different demographic and disease characteristics was analysed using logistic regression models. Stratifications included baseline demographics (child age, gender, latrine use, maternal education, maternal age, family size, family drinking water source, and living house ownership) and pre‐existing disease characteristics (IPIs, anaemia, and thinness). Possible moderating effects of each baseline variable on the effect of intervention were identified by adding interaction terms to the regression model [17]. Statistical significance was set at p < 0.05.

### Results

Baseline characteristics and the trial profile of the original project have been described previously [13]. Briefly, 365 (99%) children were analysed for six‐month follow‐up. Boys comprised 41% (n = 150) of the study participants and mean age was 10 (SD = 2.6) years. Following 6 months follow‐up, 21% (95% CI 17–25%) of the children were re‐infected with intestinal parasites, 18% (95% CI 14–22%) of the children were anaemic and 17% (95% CI 13–21%) had diarrhoea.

Table 1 describes the multivariate logistic regression analysis results of the associations between intestinal parasitosis, anaemia, and diarrhoea. Effects were adjusted for each intervention. Current intestinal parasitosis, a history of diarrhoea in the previous week, and current anaemia were independently associated. IPIs were significantly associated with anaemia (AOR 2.09, 95% CI 1.15–3.80) and diarrhoea (AOR 2.83, 95% CI 1.57–5.09), and vice versa. Anaemia and diarrhoea were also strongly related (AOR 9.62, 95% CI 5.18–17.85), meaning that children with diarrhoea had a very high chance of also having anaemia, and vice versa.

**Table 1:** Associations between intestinal parasitic infections, anaemia and diarrhoea among school-aged children, Ethiopia (n = 365)

Post-intervention out-come variables (n)	Post-intervention out-come variables		
	IPI	Anaemia	Diarrhoea
	AOR (CI)	AOR (CI)	AOR (CI)
IPI (79)	–	2.09 (1.15 to 3.80)*	2.83 (1.57 to 5.09)*
Anaemia (81)	2.09 (1.15 to 3.80)*	–	9.62 (5.18 to 17.85)*
Diarrhoea (68)	2.83 (1.57 to 5.09)*	9.62 (5.18 to 17.85)*	–

*CI* 95% confidence interval, *AOR* adjusted odds ratio as computed by the logistic regression model, *IPI* intestinal parasitic infection

* Statistically significant at 0.05

Both hand‐washing with soap (AOR 0.32, 95% CI 0.20–0.62, p = 0.001) and weekly finger nail clipping (AOR 0.51, 95% CI 0.27–0.95, p = 0.035) interventions were reported to have a significant impact in reducing intestinal parasite re‐infection rates among the study participants [13].

In this study, we explored if these impacts were similar across children with different demographic and disease backgrounds. Overall, interventions seem equally efficacious among children regardless of age, gender, drinking water source, latrine use, mother’s age, mother’s education, family size, house ownership, and history of intestinal parasitosis, anaemia and thinness at baseline (Table 2). The impact of hand‐washing was similar for the whole group and for children who had IPIs at baseline, but the effect significantly increased in children who were parasite‐free at baseline (AOR 0.48 vs. AOR 0.31, p = 0.048). The effects of hand‐washing and nail clipping were higher for children whose drinking water sources were wells and streams compared to those who used pipeline and boreholes, but the effects were not statistically significant (AOR 0.44 vs. AOR 0.08, p = 0.134 and AOR 0.68 vs. AOR 0.09, p = 0.053; respectively).

**Table 2:** Impact of hand-washing on parasite re-infection rates across children with different base-line demographic and disease characteristics (n = 365)

	Hand-washing vs. control		Nail clipping vs. control	
	0.43 (0.24, 0.68)		0.59 (0.36, 0.98)	
Gender	Male 150 (41%)	Female 215 (49%)	Male 150 (41%)	Female 215 (49%)
	AOR (CI)^a^	AOR (CI)^a^	AOR (CI)^a^	AOR (CI)^a^
	0.41 (0.17, 0.97)	0.39 (0.20, 0.75)	0.34 (0.14, 0.82)	0.79 (0.41, 1.51)
	p = 0.923		p = 0.131	
Age	6–9 years	10–15 years	6–9 years	10–15 years
	208 (57%)	157 (43%)	208 (57%)	157 (43%)
	0.30 (0.13, 0.66)	0.50 (0.25, 1.01)	0.50 (0.23, 1.09)	0.66 (0.33, 1.31)
	p = 0.338		p = 0.604	
Water source	Pipeline and borehole	Wells and streams	Pipeline and borehole	Wells and streams
	321 (88%)	44 (12%)	321 (88%)	44 (12%)
	0.44 (0.25, 0.76)	0.08 (0.01, 0.68)	0.68 (0.39, 1.17)	0.09 (0.13, 0.64)
	p = 0.134		p = 0.053	
Latrine use	Yes	No	Yes	No
	139 (38%)	226 (62%)	139 (38%)	226 (62%)
	0.33 (0.13, 0.81)	0.44 (0.23, 0.84)	0.48 (0.20, 1.19)	0.61 (0.32, 1.16)
	p = 0.744		p = 0.791	
Mother education	Literate	Illiterate	Literate	Illiterate
	161 (44%)	204 (56%)	161 (44%)	204 (56%)
	0.58 (0.26, 1.30)	0.28 (0.14, 0.58)	0.83 (0.37, 1.85)	0.42 (0.21, 0.84)
	p = 0.198		p = 0.207	
Maternal age	≤ 35	> 35	≤ 35	> 35
	193 (53%)	172 (47%)	193 (53%)	172 (47%)
	0.22 (0.09, 0.51)	0.58 (0.28, 1.22)	0.58 (0.21, 1.58)	0.71 (0.25, 2.02)
	p = 0.085		p = 0.787	
Family size	≤ 6 members	> 6 members	≤ 6 members	> 6 members
	208 (57%)	157 (43%)	208 (57%)	157 (43%)
	0.27 (0.13, 0.54)	0.69 (0.31, 1.55)	0.60 (0.30, 1.17)	0.53 (0.23, 1.20)
	p = 0.082		p = 0.814	
House ownership	Yes	No	Yes	No
	307 (84%)	58 (16%)	307 (84%)	58 (16%)
	0.45 (0.26, 0.79)	0.17 (0.03, 0.88)	0.71 (0.40, 1.24)	0.19 (0.05, 0.77)
	p = 0.661		p = 0.386	
Baseline parasite infection status	Yes	No	Yes	No
	146 (40%)	219 (60%)	146 (40%)	219 (60%)
	0.48 (0.23, 1.01)	0.31 (0.14, 0.67)	0.44 (0.20, 0.97)	0.69 (0.34, 1.43)
	p = 0.048*		p = 0.696	
Baseline anemia status	Yes	No	Yes	No
	106 (29%)	259 (71%)	106 (29%)	259 (71%)
	0.59 (0.25, 1.39)	0.32 (0.16, 0.64)	0.54 (0.23, 1.26)	0.60 (0.31, 1.15)
	p = 0.577		p = 366	
Baseline BMI-for-age	Thin	Normal	Thin	Normal
	150 (41%)	215 (59%)	150 (41%)	215 (59%)
	0.33 0.15, 0.75)	0.45 (0.23, 0.91)	0.44 (0.20, 0.97)	0.71 (0.36, 1.42)
	p = 0.577		p = 0.366	

*CI* 95% confidence interval, *AOR* adjusted odds ratio

* Statistically significant at 0.05

^a^Adjusted for intervention

### Discussion

Findings of the present study demonstrate a clear relationship between IPIs, anaemia and diarrhoea among children. Children with IPIs had a much higher chance of also being anaemic and having diarrhoea. Anaemia and diarrhoea were also very strongly related, as children with diarrhoea had a very high chance of also having anaemia, and vice versa. Hand-washing with soap and nail clipping prevents the children from intestinal parasite infections regardless their demographic differences and hence breaks the vicious cycle of IPIs, anemia and diarrhoea among the children.

Associations observed between anaemia and IPIs in our data concord with other studies that showed IPIs to be substantially linked with anaemia in children [18–21]. IPIs can decrease food and nutrient intake, cause intestinal blood losses, induce red blood cell destruction by the spleen, and induce autoimmune reactions leading to chronic inflammation [20, 21]. These effects may have accounted for the considerable proportion of anaemia observed among the children infected with intestinal parasites.

Although in most instances IPIs are asymptomatic, they may also cause diarrhea [22]. IPIs can induce diarrhoea by increasing small intestine motility while reducing its digestive and absorptive capacities [23]. Our finding that diarrhoea may contribute substantially to anaemia among children was also consistent with other reports from developing settings [24]. Diarrhoeal diseases are reported to be associated with an increased production of cytokines, interleukin 6 and tumour necrosis factor alpha [25]. These cytokines are indicated to play a significant role in causing anaemia [24]. Repeated episodes of diarrhoea in children are also reported to lead to decreased nutrient absorption, due to injury of the small intestine mucosa [26]. In our data, anaemia was also an independent risk factor for both IPIs and diarrhoea. In agreement with our findings, Levy et al. [27] have reported that anaemia increases rates of infection in children. Furthermore, reports from several studies have indicated that anaemia can predispose people to infections by lowering host immunity [28, 29]. In general, our findings strengthen the well‐established notion that infection and malnutrition are intricately linked [1, 30, 31].

The observed significant preventive impact of hand‐washing and nail clipping on intestinal parasite re‐infection rates made us curious to explore whether the intervention effect noted in the whole cohort was homogenous across children with different backgrounds. Hand‐washing with soap and nail clipping interventions consistently favoured reduction of intestinal parasite re‐infection rates across each sub‐group analysed. A significant difference in the effect of hand‐washing was observed only for baseline parasitic infection status. Based on our data, it is possible to suggest that benefit from the interventions is likely to be more universal among the study groups. The observed increased benefit of hand‐washing among children who were parasite‐free at baseline should be interpreted with caution, although analyses were based on formal tests of interaction. Children were made parasite‐free at recruitment and this might have affected our analysis at follow‐up. The difference in effect for hand‐washing and nail clipping between children who use pipeline and borehole water sources and those using wells and streams is quite large, but not significant because of the small number of children who use wells and streams.

In conclusion, our findings emphasise that intestinal parasitosis, anaemia and diarrhoea were independently associated. Furthermore, hand‐washing with soap and nail clipping were efficacious in preventing intestinal parasite re‐infection despite baseline differences and hence can be universally used as infection prevention interventions among school‐aged children to break the vicious cycle of infection and malnutrition for the long‐term health benefits of this population.

## Limitations

The following limitation should be considered when interpreting the results of the present study: the study was powered to determine the overall effect of the interventions in the original randomized controlled trial. Our subgroup analyses might hence be underpowered to detect subgroup effects, unless the differences in treatment effects between subgroups would have been very large.

## Data Availability

The datasets used and/or analysed during the current study are available from the corresponding author on reasonable request.

## Abbreviations

AOR: adjusted odds ratio
BMI: body-mass-index
CI: confidence interval
IPIs: intestinal parasitic infections
OR: odds ratio
SD: standard deviation
WHO: World Health Organization
